# Association between CCR2 and CCL2 expression and NET stimulation in adult-onset Still’s disease

**DOI:** 10.1038/s41598-023-39517-4

**Published:** 2023-07-27

**Authors:** Ju-Yang Jung, Mi-Hyun Ahn, Ji-Won Kim, Chang-Hee Suh, Jae Ho Han, Hyoun-Ah Kim

**Affiliations:** 1 Department of Rheumatology, Ajou University School of Medicine, 164, World Cup-ro, Yeongtong-gu, Suwon, 16499 Republic of Korea; 2 Department of Pathology, Ajou University School of Medicine, 164, World Cup-ro, Yeongtong-gu, Suwon, 16499 Republic of Korea

**Keywords:** Molecular biology, Rheumatology

## Abstract

Adult-onset Still’s disease (AOSD) is a systemic inflammatory disease characterized by the activation of monocyte-derived cells and the release of neutrophil extracellular traps (NET). C–C motif ligand (CCL) 2 is a chemoattractant that interacts with the C–C motif chemokine receptor (CCR) 2, resulting in monocyte recruitment and activation. CCL2 and CCR2 were measured with enzyme-linked immunosorbent assay (ELISA) at the serum level, and using immunohistochemical staining at the skin and lymph node tissues levels. THP-1 cell lysates were analyzed using western blot and ELISA after NET stimulation in patients with AOSD. Serum CCL2 level was higher in patients with AOSD than in patients with rheumatoid arthritis and healthy controls (HCs). In patients with AOSD, the percentage of CCL2-positive inflammatory cells in the skin tissues and CCR2-positive inflammatory cells in the lymph nodes increased, compared to that in HCs and in patients with reactive lymphadenopathy, respectively. NET induced in patients with AOSD enhanced the secretion of CCR2, higher CCR2 expression in monocytes, and the levels of interleukin (IL)-1β, IL-6, and IL-18 from THP-1 cells. Our findings suggest that upregulation of the CCL2–CCR2 axis may contribute to the clinical and inflammatory characteristics of AOSD.

## Introduction

Chemokines are small-sized chemotactic cytokines (8–14 kDa) that modulate leukocyte movement in organ development, homeostasis, angiogenesis, and immune response^1,2^. Released by recruited monocytes and monocyte-derived cells, these molecules play an important role in facilitating leukocyte homing and trafficking in inflammatory sites^3^. Among CC chemokines, C–C motif ligand (CCL) 2, also known as monocyte chemoattractant protein-1 (MCP-1), is a chemoattractant for mononuclear cells and inflammatory tissues, whose expression is triggered by inflammatory stimuli. CCL2 typically binds to C–C motif chemokine receptors (CCR) 2, which is highly expressed in monocytes and macrophages. This binding activates the signaling cascades, thereby, leading to cell migration and infiltration into tissues^4,5^.

Adult-onset Still's disease (AOSD) is a rare systemic inflammatory disease that is characterized by high spiking fever along with systemic features including evanescent rash, generalized lymphadenopathy, serositis, and arthritis^6^. Although the pathogenesis of AOSD remains unclear and unknown, several etiologic factors such as infections, genetic backgrounds, and immune dysregulation contribute to the development of the disease^6,7^. Several pathogen-associated molecular patterns or damage-associated molecular patterns (DAMPs) trigger innate immune activation, including macrophage activation with upregulated macrophage-related mediators^8,9^. Interleukin (IL)-1β, IL-6, IL-18, IL-8, and interferon (IFN)-γ-induced chemokines are high in patients with AOSD, suggesting that proinflammatory cytokines and chemokines play important roles in their immune dysregulation^10–12^. In addition, several chemokines, such as CXCL8, CXCL10, and CXCL12, serve as biomarkers for predicting disease activity or cause persistence of arthritis in patients with AOSD^12–15^.

Neutrophil extracellular traps (NET) are formed by dying neutrophils and act as a DAMP, which can activate macrophages to secrete proinflammatory cytokines and chemokines^16,17^. The death of neutrophils with NET formation, a process that is referred to as NETosis, leads to the expression of some signals on the cell surface via trapped macrophages^18^. Among classically (M1) and alternatively (M2) activated macrophages, M2 macrophages promote an anti-inflammatory response, whereas M1 macrophages induce cell death in neutrophils, leading to the release of extracellular DNAs called NETosis. According to reports, NET formation was increased in the serum and tissues of patients with AOSD^19,20^. Additionally, neutrophils obtained from patients with active AOSD released NETs that contained enhanced levels of IL-1β upon stimulation^21^. Moreover, the extent of NET formation was correlated with disease activity, the presence of arthritis, fever, cutaneous manifestations, and response to glucocorticoids in patients with AOSD^20,22^. The activation of M1 macrophage is involved in the process of NET formation, leading to the release of elevated levels of ferritin, a useful biomarker for diagnosing and assessing disease activity in AOSD^23^.

The CCR2-CCL2 axis, which is involved in monocyte/macrophage activation and NET formation, contributes to proinflammatory conditions^5^. In the context of influenza infection, neutrophils recruited to the lung express various chemokine receptors, including CCR2, and the activation of these chemokine receptors triggers NET formation^24^. Furthermore, in patients with myocardial infarction, NETs and levels of CCL2 increased at the site of injury, and stimulation of NETs led to the secretion of CCL2 and downregulation of CCR2^25^. Notably, while CCL2 promoted NET formation in neutrophils from healthy donors in vitro, the expression and roles of chemokines and their receptors may differ in specific diseases due to variations in pathological pathways^25^.

While increased levels of inflammatory cytokines and NET formation play a role in the pathogenic mechanisms of AOSD, research on the levels of chemokine receptors and their impact on the NET formation is limited, specifically in the context of AOSD. Therefore, the levels of CCL2 and CCR2, the associations between their expression level, and the disease activity or clinical manifestation in patients with AOSD were evaluated in this study. In addition, we aimed to find an interaction between the CCL2-CCR2 axis and NETosis and determine whether the inflammation triggered by NETosis could be alleviated if CCR2 is blocked.

## Results

### Clinical characteristics of subjects

Supplementary Table S1 summarizes the clinical characteristics of the subjects. The mean age of patients with AOSD was 44.3 ± 14.2 years, and 35 (83.3%) patients were female. Age and sex were matched among the patients with AOSD and rheumatoid arthritis (RA) as well as healthy controls (HCs). Thirty-one patients had active disease, while 30 (71.4%), 27 (64.3%), and 23 (54.8%) patients had fever, skin rashes, and arthralgia, respectively. The systemic score was 3.48 ± 2.43 in patients with AOSD, and the disease activity score including 28 joints was 2.72 ± 1.37 in patients with RA. Patients with AOSD also had higher levels of erythrocyte sedimentation rate (ESR), C-reactive protein (CRP), ferritin, lactate dehydrogenase (LDH), aspartate transaminase (AST), and alanine transaminase as compared to patients with RA.

### Comparisons of CCL2 and CCR2 serum levels in HCs and patients with AOSD and RA

**Figure 1:**
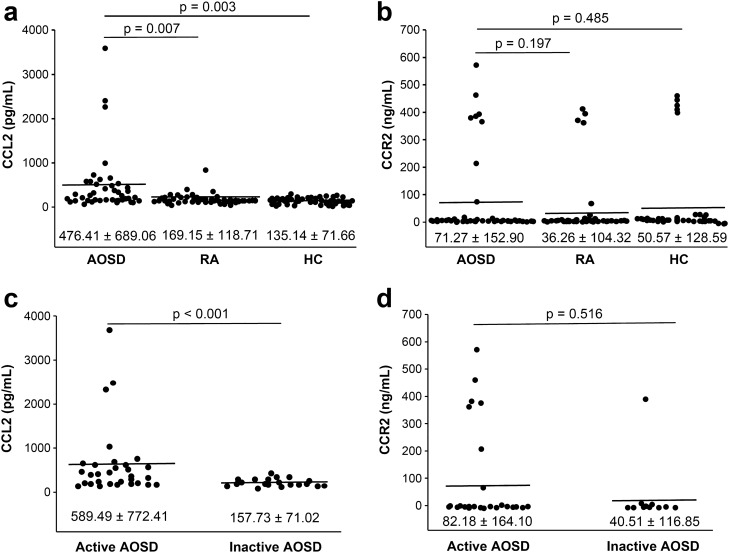
Serum CCL2 (**a**) and CCR2 (**b**) levels in patients with AOSD and RA, and HCs. Serum CCL2 (**c**) and CCR2 (**d**) levels in patients with active and inactive AOSD. *CCR* C–C motif chemokine receptor, *CCL* C–C motif ligand, *AOSD* adult-onset Still’s disease, *RA* rheumatoid arthritis, *HCs* healthy controls.

Serum CCL2 levels were significantly increased in patients with AOSD (476.41 ± 689.06 pg/mL) compared to that in patients with RA (169.15 ± 118.71 pg/mL, *p* = 0.007) and in HCs (135.14 ± 71.66 pg/mL, *p* = 0.003) (Fig. 1a, b). Patients with AOSD had higher serum CCR2 levels (71.27 ± 152.9 ng/mL) than patients with RA (36.26 ± 104.32 ng/mL, *p* = 0.197) and HCs (50.57 ± 128.59 ng/mL, *p* = 0.485). However, these differences were not statistically significant due to the large variation between individuals.

### Serum CCL2 and CCR2 levels according to disease activity of AOSD

Serum CCL2 levels were significantly higher in patients with active AOSD (589.49 ± 772.41 pg/mL) as compared to those with inactive AOSD (157.73 ± 71.02 pg/mL, *p* < 0.001) (Fig. 1c, d). Serum CCR2 levels were higher in patients with active AOSD (82.18 ± 164.1 ng/mL) compared to those with inactive AOSD; however, this difference was not statistically significant (40.51 ± 116.85 ng/mL, p = 0.516).

The correlations between disease activity markers and the serum levels of CCL2 or CCR2 in patients with AOSD are shown in Table 1. Serum CCL2 levels correlated with systemic scores (r = 0.539, *p* < 0.001), leukocyte counts (r = 0.316, *p* = 0.041), neutrophil counts (r = 0.316, p = 0.041), CRP (r = 0.312, *p* = 0.044), ferritin (r = 0.607, *p* < 0.001), LDH (r = 0.597, *p* < 0.001), and albumin (r = − 0.428, *p* = 0.005) in patients with AOSD. However, the serum CCR2 levels were not associated with any disease activity markers of AOSD.

**Table 1:** Correlation between disease activity markers and CCR2/CCL2 in patients with adult-onset Still’s disease.

Disease activity markers	Correlation coefficient, r (*p*-value)	
	CCR2	CCL2
Systemic score	− 0.034 (0.832)	0.539 (< 0.001)
Hemoglobin	0.109 (0.491)	0.27 (0.083)
Leukocyte	0.095 (0.551)	0.316 (0.041)
Neutrophil	0.072 (0.65)	0.316 (0.041)
Platelet	− 0.012 (0.941)	− 0.316 (0.041)
ESR	− 0.003 (0.985)	0.262 (0.094)
CRP	0.12 (0.447)	0.312 (0.044)
Ferritin	0.103 (0.516)	0.607 (< 0.001)
LDH	0.001 (0.993)	0.597 (< 0.001)
Albumin	− 0.057 (0.719)	− 0.428 (0.005)
Bilirubin	0.093 (0.561)	− 0.134 (0.405)
AST	0.021 (0.894)	0.372 (0.015)
ALT	0.061 (0.7)	0.301 (0.052)
CCR2	–	0.126 (0.425)
CCL2	0.126 (0.425)	–

*CCR* C–C motif chemokine receptor, *CCL* C–C motif ligand, *ESR* erythrocyte sedimentation rate, *CRP* C-reactive protein, *LDH* lactate dehydrogenase, *AST* aspartate transaminase, *ALT* alanine transaminase.

Linear regression analysis showed that serum CCR2 levels were associated with leukocyte count (*β* = 0.417 *p* < 0.001), ESR (*β* = 0.239, *p* = 0.016), and CRP (*β* = 0.27, *p* = 0.006) (Supplementary Table S2). Serum CCL2 levels were associated with systemic scores (*β* = 0.316, *p* = 0.024), hemoglobin (*β* = − 0.236, *p* = 0.018), leukocyte counts (*β* = 0.316, *p* = 0.041), ESR (*β* = 0.242, *p* = 0.015), ferritin (r = 0.437, *p* = 0.001), and AST (*β* = 0.225, *p* = 0.024) in patients with AOSD.

### Serum CCL2 or CCR2 levels according to disease manifestations of AOSD and change of serum levels after resolution of disease

A comparison was made between the serum CCR2 and CCL2 levels in patients with specific manifestations of AOSD and those without the manifestations (Supplementary Table S3). The results indicate no significant differences in the serum CCR2 levels between the two groups. CCL2 levels were significantly higher in patients with AOSD who had a fever (591.95 ± 786.11 pg/mL) than in those without fever (187.55 ± 114.44 pg/mL, *p* = 0.003). CCL2 levels were also significantly higher in those with skin rash (550.61 ± 755.35 pg/mL) than in those without skin rash (342.84 ± 548.78 pg/mL, *p* = 0.019).

Linear regression analysis showed an association between CCL2 levels and fever (*β* = 0.304, p = 0.03). However, no significant association was found between CCR2 levels and any of the manifestations analyzed in the study (Supplementary Table S2).

Serum CCL2 levels decreased significantly (472.46 ± 280.11 to 223.85 ± 53.36 pg/mL, *p* = 0.029) in seven patients with AOSD after the clinical profiles improved (Supplementary Table S4). However, serum CCR2 levels did not differ after improvement in symptoms of AOSD.

### Comparisons of CCL2/CCR2 deposits in the skin and lymph nodes of patients with AOSD

**Figure 2:**
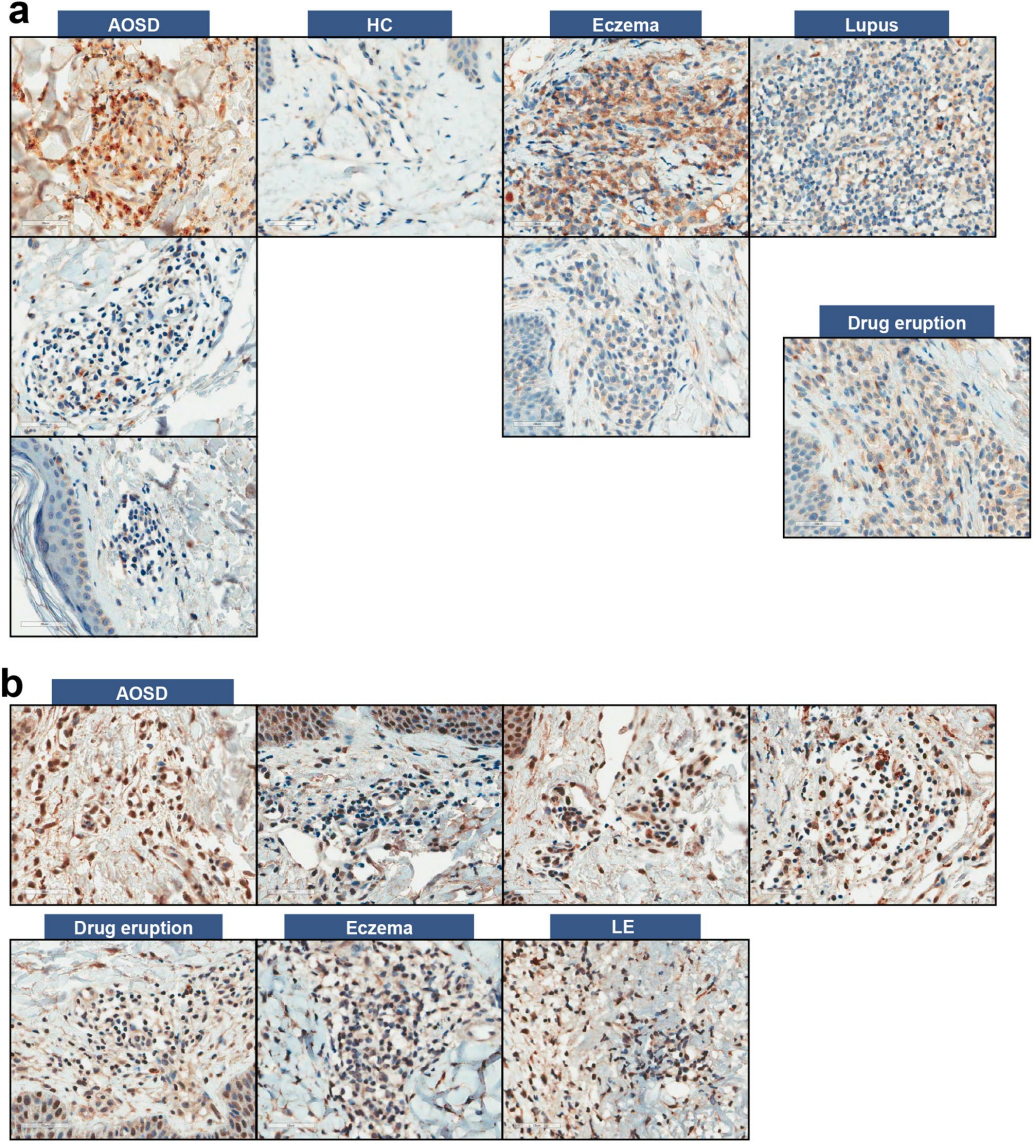
Immunostaining results of CCL2 and CCR2 in skin tissues from 35 patients with AOSD, five HCs, five eczema patients, five lupus patients, and five drug eruption patients. (**a**) CCL2-stained cells and (**b**) CCR2-stained cells.

Immunohistochemical (IHC) findings of the skin tissues and lymph nodes of patients with AOSD are shown in Table 2. For skin tissues, CCL2-stained cell proportions in patients with AOSD (n = 35, 5.3 ± 7.8%) were higher compared to those in HCs (n = 5, 0.6 ± 0.5%, *p* = 0.03) but were similar to that in patients with eczema (n = 5, 3.4 ± 1.5%, *p* = 0.43), systemic lupus erythematosus (SLE, n = 5, 2.0 ± 1.0%, *p* = 0.78), and drug eruption (n = 5, 7.0 ± 12.9%, *p* = 0.84) (Fig. 2). In addition, CCR2-stained cell proportions in patients with AOSD (24.7 ± 21.2%) were similar to that in HCs (26.2 ± 23.5%, *p* = 0.84) and patients with SLE (22.2 ± 29.1%, *p* = 0.317); however, these proportions were higher than that in patients with eczema (5.2 ± 3.2%, *p* = 0.01) and drug eruption (6.2 ± 3.6, *p* = 0.01).

**Table 2:** Immunostaining for CCL2 and CCR2 in the skin and lymph nodes.

Staining cell proportion in skin tissue, %	AOSD (n = 35)	HCs (n = 5)	Eczema (n = 5)	Lupus (n = 5)	Drug eruption (n = 5)
CCL2	5.3 ± 7.8	0.6 ± 0.5	3.4 ± 1.5	2.0 ± 1.0	7.0 ± 12.9
*p-*value (vs. AOSD)	–	0.03	0.43	0.78	0.84
CCR2	24.7 ± 21.2	26.2 ± 23.5	5.2 ± 3.2	22.2 ± 29.1	6.2 ± 3.6
*p-*value (vs. AOSD)	–	0.84	0.01	0.32	0.01

Staining cell proportion in lymph nodes, %	AOSD (n = 9)	Tuberculosis lymphadenitis (n = 5)	T cell lymphoma (n = 5)	Kikuchi’s disease (n = 5)	Reactive lymphadenopathy (n = 5)
CCL2	4.6 ± 6.0	16.0 ± 12.9	5.2 ± 3.6	9.6 ± 11.9	3.0 ± 2.9
*p-*value (vs. AOSD)		0.01	0.31	0.44	0.68
CCR2	26.8 ± 24.9	4.2 ± 3.6	6.4 ± 7.9	7.8 ± 7.6	4.8 ± 3.3
*p-*value (vs. AOSD)	–	0.02	0.06	0.1	0.04

*AOSD* adult-onset Still's disease, *HCs* healthy controls, *CCR* C–C motif chemokine receptor, *CCL* C–C motif ligand.

**Figure 3:**
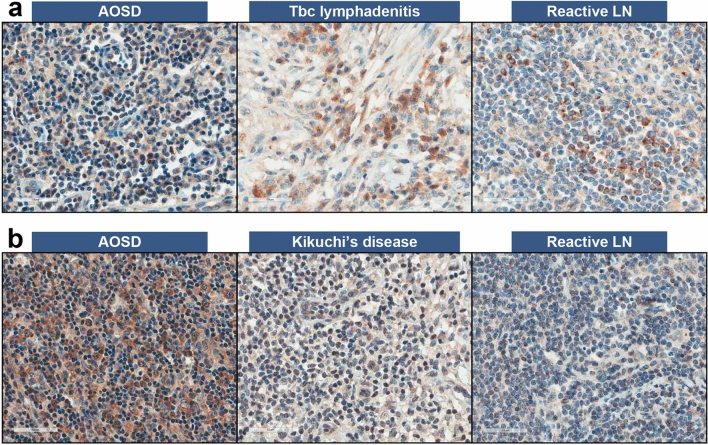
Immunostaining results of CCL2 and CCR2 in lymph nodes from nine patients with AOSD, five tuberculosis lymphadenitis, five T cell lymphoma, five Kikuchi’s disease, and five Reactive LN. (**a**) CCL2-stained cells, and (**b**) CCR2-stained cells. *LN* lymphadenopathy.

For lymph nodes, CCL2-stained cell proportions in patients with AOSD (n = 9, 4.6 ± 6.0%) were lower compared to that in patients with tuberculosis lymphadenitis (n = 5, 16.0 ± 12.9%, *p* = 0.01), but were similar to the proportions in patients with T cell lymphoma (n = 5, 5.2 ± 3.6%, *p* = 0.31), Kikuchi’s disease (n = 5, 9.6 ± 11.9%, *p* = 0.44), and reactive lymphadenopathy (n = 5, 3.0 ± 2.9%, *p* = 0.68) (Fig. 3). In addition, CCR2-stained cell proportions in patients with AOSD (26.8 ± 24.9%) were higher than that in patients with tuberculosis lymphadenitis (4.2 ± 3.6%, *p* = 0.02) and reactive lymphadenopathy (4.8 ± 3.3%, *p* = 0.04) but were similar to that in patients with T cell lymphoma (6.4 ± 7.9%, *p* = 0.06) and Kikuchi’s disease (7.8 ± 7.6%, *p* = 0.1).

### Effect of NETs on inflammatory cytokines levels and intracellular transcription through CCR2 involvement in AOSD

**Figure 4:**
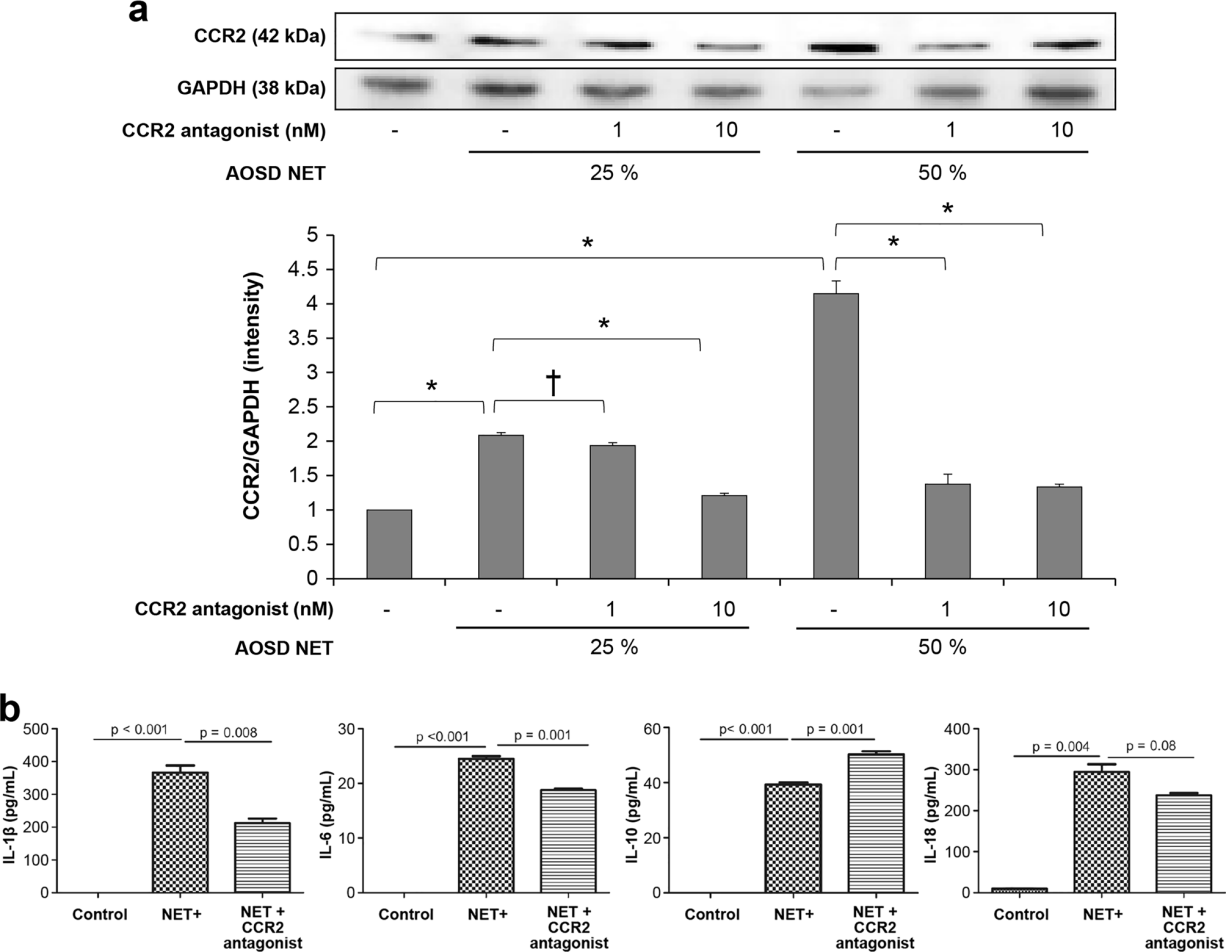
Changes in CCR2 and inflammatory cytokines according to the treatment of NETs from patients with AOSD. (**a**) Expression of CCR2 after treatment of NET contents and CCR2 antagonist on THP-1 cell. AOSD NETs percentage: amounts of culture media (*p < 0.01, ^†^*p* < 0.05). (**b**) Levels of IL-1β, IL-6, IL-10 and IL-18 after treatment of NET contents and CCR2 antagonist on THP-1 cell. *NET* neutrophil extracellular traps, *IL* interleukin.

CCR2 expression on THP-1 cells increased after the administration of NET, which is composed of 25% and 50% of culture media (both *p* < 0.01) (Fig. 4). In contrast, CCR2 expression was decreased after the administration of CCR2 antagonist treatment (1 nM, *p* < 0.05 in AOSD NETs 25%, and *p* < 0.01 in AOSD NETs 50%; 10 nM, both *p* < 0.01 in AOSD NETs 25% and 50%).

The levels of IL-1β (365.9 ± 38.7 pg/mL) and IL-6 (24.5 ± 0.9 pg/mL) increased after the administration of NET contents on THP-1 cells (*p* < 0.001). After the administration of the CCR2 antagonist, the levels of IL-1β (212.8 ± 23.1 pg/mL, *p* = 0.008) and IL-6 (18.8 ± 0.4 pg/mL, *p* = 0.001) decreased. Levels of IL-10 decreased (39.3 ± 1.2 pg/mL) after administration of NET contents on THP-1 cells (*p* < 0.001) and increased after administration of CCR2 antagonist (50.3 ± 2.0 pg/mL, *p* = 0.001). Further, levels of IL-18 increased (294.9 ± 31.8 pg/mL) after the administration of NET contents on THP-1 cell (*p* = 0.004) but did not decrease after the administration of CCR2 antagonist (237.9 ± 8.9 pg/mL, *p* = 0.08).

Furthermore, the downstream signaling pathway of the CCL2-CCR2 axis was investigated through the stimulation of AOSD NETs. Phosphorylation of JNK (p-JNK) and NF-κB (p- NF-κB) was increased upon administration of AOSD NETs at 50% and AOSD NETs at 25%, and the levels of p-JNK and p-NF-κB significantly decreased upon administration of CCR2 antagonist (Supplementary Fig. S1).

## Discussion

This study evaluated the serum CCL2 and CCR2 levels in patients with AOSD and their potential associations with disease activity and clinical manifestations of AOSD. Additionally, the study investigated the interactions between CCR2 and NET formation in AOSD, along with the change in levels of inflammatory cytokines.

CCL2 has chemotactic effects on immune cells; however, CCL2 also influences myeloid cell behavior, including enhanced macrophage survival, proliferation, cytotoxicity, phagocytic ability, and M2 polarization^5^. CCL2 expression is triggered by lipopolysaccharides via IL-1β and IL-6^27^. Further, CCL2 induces the production of proinflammatory cytokines including IL-6 and IL-1β from monocytes and macrophages^26–30^. In this study, serum CCL2 levels were increased in patients with AOSD and correlated with disease activity markers including systemic score, leukocyte and neutrophil count, CRP, and ferritin levels. Furthermore, CCL2 levels were lower after disease remission, as the levels of cytokines, including IL-1β and IL-6, were lower in disease remission. In addition, groups with fever or skin rash had higher levels of CCL2 than those without the rash. Other studies have revealed that patients with systemic juvenile idiopathic arthritis (JIA) had higher levels of CCL2 as compared to HCs, and the levels of CCL2 were associated with the current systemic features of systemic JIA^31–33^. A comparison of multiple cytokine signatures in JIA revealed that plasma levels of tumor necrosis factor (TNF)-α, which is a macrophage inhibitory factor, as well as CCL2, CCL3, CCL11, CCL22, and CXCL9, were elevated in patients with JIA; however, CCL2 levels were not significantly different in patients with systemic JIA as compared to those with other subtypes of JIA^13,34^. In another study concerning AOSD, the serum CCL2 levels were higher in patients with AOSD as compared to HCs with some other chemokines^13^. Those findings identified that the serum CCL2 levels represented the severity of systemic inflammation and might be a promising biomarker for AOSD.

Previous reports have shown that the CCL2-CCR2 axis is enhanced in RA^35,36^. These studies found increased CCL2 expression in the synovium, leading to enhanced IL-6 and IL-8 production by fibroblast-like synoviocytes in patients with RA; however, serum CCL2 levels decreased after TNF blockade in these patients^35,36^. Neutrophils expressing CCR2 were increased, and the chemotactic effect of CCL2 was enhanced in the peripheral blood of patients with RA^37^. However, in this study, serum CCL2 levels did not increase in patients with RA with mild disease activity, as indicated by the supplementary data S1 (DAS28 2.72 ± 1.37). Therefore, it can be concluded that serum CCL2 levels might be increased due to immunopathologic response in both AOSD and RA and downregulated by controlling inflammation in both diseases. Markedly raised levels of CCR2, which chemoattracts CCL2 and promotes inflammation in the synovium of RA, along with the increased proportions of CCL2 expression in the skin and the lymph nodes, suggest that the CCL2/CCR2 axis enhances tissue inflammation in AOSD. In addition, IHC studies have revealed several findings in patients with AOSD^15,38,39^. Lymph nodes of patients with AOSD exhibited paracortical hyperplasia, infiltration of reactive lymphocytes, and inflammatory cells, along with a higher expression of CXCL10 and CXCL13^38,40^. In skin lesions of patients with AOSD, an elevated proportion of inflammatory cells expressing IL-33, epidermal keratinocytes with phosphorylated signal transducer and activator of transcription 3 (STAT3)-positive nuclei was observed^41,42^. These findings suggest that the target lesions in AOSD have distinctive characteristics, such as the enhanced proliferation and survival of inflammatory cells and the involvement of chemokines. Furthermore, the skin and lymph nodes of patients with AOSD showed the presence of neutrophil elastase-positive and myeloperoxidase (MPO)-positive inflammatory cells, indicating the occurrence of NET formations^21^. An association between the expression of these chemokines and MPO and the expression of CCL2 and CCR2 in the skin and lymph nodes is being speculated.

In a previous study, the skin tissues of patients with AOSD showed inflammatory cell infiltration with keratinocyte necrosis^39^. Stimulated keratinocytes express CCL2 in inflammatory skin reactions, and the activation of mononuclear cells and severity of skin lesions have been associated with CCL2 concentration in patients with psoriasis^43,44^. CCL2- or CCR2-depletion attenuated neutrophil accumulation and IL-1β expression in the skin of irritant contact dermatitis mice models^45^. Our study showed that CCL2 deposits in skin tissues were significantly increased in patients with AOSD, suggesting that CCL2 might be associated with monocyte infiltration in the cutaneous manifestation of AOSD. Moreover, inhibition of CCL2 deposits might be considered a treatment option in some patients who suffer from exacerbated skin symptoms, such as rash, irritation, and itching, during the chronic course of AOSD.

Peripheral blood monocytes express particular chemokine receptors depending on the specific stimulus or condition, and monocytes expressing CCR2 are recruited to the inflamed tissue with strong proinflammatory functions^46^. The CCL2-CCR2 axis drives the trafficking of blood monocytes into the inflamed tissue, and CCR2 deficiency reduces the recruitment of monocytes^47^. In addition, CCR2 expression is induced by proinflammatory cytokines including IFN signatures and TNF-α in inflamed tissues but not in the peripheral blood^48^. The present study revealed that the serum CCR2 levels were not significantly different in patients with AOSD and did not show any correlation with disease activity markers or clinical manifestations. However, CCR2 deposition in lymph nodes was increased in patients with AOSD as compared to several other diseases, including tuberculosis lymphadenitis, T cell lymphoma, and Kikuchi’s disease. These findings suggest that the expression of CCR2 on neutrophils or lymphocytes within the tissue may impact the inflammatory response in patients with AOSD, while serum CCR2 levels might not play a significant role in these patients.

NET formation can be considered one of the end results of macrophage activation. NET functions as a DAMP itself and can promote an immune response in patients with AOSD^49^. NET, which was derived from patients with RA, stimulated the fibroblast-like synoviocytes to release IL-6 and IL-8^50^. The present study revealed that NET, derived from patients with AOSD, stimulated monocytes to promote CCR2 expression. Considering that NET formation is enhanced in AOSD^21^, the interaction between NET and CCR2 could have an influence on monocyte infiltration into targeted tissues. This finding, for the first time, reveals the effect of AOSD NET on chemokine expression, suggesting a critical role of NET formation in the increased expression of proinflammatory chemokines and monocyte recruitment in patients with AOSD. Furthermore, we observed the phosphorylation of the intracellular transcription factors, JNK and NF-κB, which were activated upon stimulation of AOSD NETs. Importantly, when CCR2 was inhibited, it attenuated the increased phosphorylation of JNK and NF-κB, suggesting that the CCL2-CCR2 axis plays a role in activating the JNK and NF-κB signaling pathways in response to AOSD NETs. CCL2 can stimulate monocyte and macrophage to produce cytokines, such as IL-1β and IL-6, and proinflammatory cytokines that can stimulate mononuclear cells^28^. Patients with systemic JIA and AOSD had higher serum concentrations of IL-1β, IL-18, and IL-6, which were derived from activated mononuclear cells^10,11,51^. In addition, CCL2 might trigger a vicious cycle that exacerbates and maintains immune responses, including activated monocyte-derived cells and abundant proinflammatory cytokines in patients with AOSD. It should be considered that the effect of CCL2 on inflammation is limited. Although the expression of CCL2–CCR2 increased in inflamed synovial tissues or synoviocytes of patients with RA, blocking the CCL2–CCR2 axis failed to make a clinical or IHC improvement in RA^52–54^. Since critical chemokines in systemic inflammatory diseases, including AOSD and RA, interact with other signaling pathways, it might be insufficient to target such chemokines or their receptors.

This study had several limitations that should be acknowledged. Firstly, AOSD is a rare disease, resulting in a small sample size of follow-up patients. Therefore, analysis of the association of the CCL2–CCR2 axis with disease outcomes or prognosis of AOSD was not possible. Secondly, the study findings indicated that serum CCL2 levels were indicative of active disease and suggested the involvement of represented active disease and the CCL2-CCR2 axis might contribute to systemic inflammation and skin rash in these patients. Third, the study did not include febrile diseases, such as SLE or vasculitis, as positive controls to compare and validate the findings.

In conclusion, this study revealed that serum CCL2 levels were elevated in patients with AOSD as compared to HCs and that these levels correlated with disease activity markers for AOSD. Patients with AOSD experiencing fever or skin rash exhibited higher levels of CCL2. Furthermore, CCL2 deposits in skin tissues and CCR2 deposits in lymph nodes were increased in patients with AOSD. The formation of NETs, which are induced in the serum of patients with AOSD, resulted in increased levels of CCR2 from monocytes. These findings suggest that upregulation of the CCL2-CCR2 axis may contribute to the clinical and inflammatory characteristics of AOSD.

## Methods

### Subjects

This study enrolled patients who were diagnosed with AOSD according to Yamaguchi’s criteria as well as age- and sex-matched HCs, who did not have malignancy, infection, and autoimmune diseases^55^. In addition, age- and sex-matched patients with RA, who met the 1987 American College of Rheumatology classification criteria, were also recruited^56^. They were enrolled in the Rheumatology Clinic of the Ajou University Hospital between January 2012 and December 2020. Their clinical dataset included their medical histories, clinical manifestations, physical examinations, and laboratory findings of the subjects. Skin tissues were obtained from patients with AOSD, eczema, lupus, and drug eruption. Skin tissues that were confirmed to have normal histology were used as healthy control samples. Lymph node tissues were obtained from patients with AOSD, tuberculosis lymphadenitis, T cell lymphoma, Kikuchi’s disease, and reactive lymphadenitis. These diseases were selected, as these diseases share pathological features with the skin lesion and lymph nodes observed in patients with AOSD. For example, eczema, skin lesions in SLE, and drug eruption exhibit characteristics such as inflammatory cell infiltration, damage to normal skin tissues, and visible eruptions. These inflammatory lesions were utilized as control groups to specifically investigate the impact of the CCL2–CCR2 axis on inflammation in patients with AOSD. All the subjects provided informed consent to participate in this study, which was conducted in accordance with the principles of the Declaration of Helsinki. The study protocol was reviewed and approved by the Ajou University Hospital Institutional Review Board (AJIRB-BMR-KSP-20-159).

### Measurement of secreted CCR2 and CCL2 in the serum by ELISA

Serum was collected from 42 patients with AOSD, 50 patients with RA, and 49 HCs. Commercial enzyme-linked immunosorbent assay (ELISA) kits (LSBio, Seattle, WA) were used for the quantification of CCL2 and secreted CCR2 in serum.

### IHC staining of the lymph node and skin of AOSD patients

All the tissues (skin and lymph node) were washed with phosphate-buffered solution (PBS), fixed with 4% paraformaldehyde in PBS for 1 h at room temperature, dehydrated with xylene, and embedded in a paraffin block. After blocking the paraffin-embedded slide with serum buffer, it was incubated with anti-CCL2 antibody and anti-CCR2 antibody (both 1:100, R&D Systems, Minneapolis, MN) at 4 °C overnight. After washing, the slides were incubated with HRP-linked anti-goat IgG secondary antibody (GBI Labs, Bothell, WA). The slides were washed and visualized using 3,3′-diaminobenzidine substrate (Merck, Darmstadt, Germany).

### THP-1 monocyte cell line culture and NET stimulation

Cells from THP-1, a human monocyte cell line, were cultured in a RPMI 1640 medium (Welgene Inc., Gyeongsan, Republic of Korea) with 10% fetal bovine serum (Thermo Fisher Sci., Waltham, MA) and 1% antibiotics (penicillin and streptomycin; Welgene Inc., Gyeongsan, Republic of Korea). After the cells were stabilized, 1 or 10 nM of CCR2 antagonist (Invivogen, San Diego, CA) was added for pre-treatment for 1 h. A medium containing a certain concentration of NET without growth factor was added and cultured for 24 h. NETs were prepared by culturing neutrophils with phorbol myristate acetate stimulation from patients with AOSD as described in our previous report^21^.

### Confirmation of protein expression by western blot

The total protein from THP-1 cell lysate stimulated with NETs was extracted using RIPA lysis buffer (Thermo Fisher Scientific., Waltham, MA) with a protease inhibitor (Thermo Fisher Scientific., Waltham, MA). Then, 20 µg of total protein was run on the sodium dodecyl sulfate polyacrylamide gel (SMOBIO, Hsinchu, Taiwan) to separate the protein, and it was transferred to a polyvinylidene difluoride membrane (Bio-Rad, Hercules, CA). This membrane was incubated with an anti-CCR2 antibody (1:1000, Cell Signaling Tech., Danvers, MA) to determine the amount of CCR2 protein, and visualized with HRP-linked anti-goat IgG (1:10,000, Abcam, Cambridge, UK). Supplementary Fig. S2 displays the western blot original file.

### Inflammatory cytokine measurement from the supernatant of the cultured cells

Cytokine concentrations (IL-1β, IL-6, IL-10, and IL-18) from the supernatant of the cultured cells were measured using commercial ELISA kits (R&D Systems, Inc., Minneapolis, MN) according to the manufacturer’s protocol.

### Statistical analysis

All data were presented as the mean ± standard deviation. CCR2 and CCL2 levels in patients with AOSD and RA, as well as the HCs, were compared using an independent *t*-test or one-way ANOVA (analysis of variance) test. Spearman's correlation and linear regression analysis were performed to evaluate the associations between the levels of CCL2 and CCR2 with disease activity markers or clinical manifestations in AOSD. CCR2 and CCL2 levels in patients with active or inactive AOSD were compared using the Mann–Whitney U test. Wilcoxon’s signed-rank test was used to compare the chemokine levels in patients who underwent follow-up serum sampling. All statistical analyses were performed using SPSS version 23.0 (SPSS Inc., Chicago, IL). In all analyses, *p* < 0.05 was set to indicate statistical significance.

### Ethics declarations and approval for human experiments

The study was approved by the Institutional Review Board of Ajou University Hospital (AJIRB-BMR-KSP-20-159).

### Consent to participate

All study participants provided informed consent.

## Supplementary Information

Supplementary Information.

## Data Availability

All data are available in the manuscript and supplementary file S1.
