# Supplementary material for: Association between CCR2 and CCL2 expression and NET stimulation in adult-onset Still’s disease
Source: Sci Rep. 2023 Jul 27;13:12218. doi: 10.1038/s41598-023-39517-4 (PMC10374521; doi:10.1038/s41598-023-39517-4)

Supplementary Table 1. Clinical characteristics of the subjects.

	AOSD (n = 42)	RA (n= 50)	HC (n = 49)
Age (year)*	44.3 ± 14.2	45.6 ± 8.6	40.6 ± 8.6
Sex (F/M)*	35/7	43/7	42/7
Active/Inactive	31/11		
Fever (%)	30 (71.4)		
Sore throat	14 (33.3)		
Skin rash	27 (64.3)		
Lymphadenopathy	10 (23.8)		
Splenomegaly	4 (9.5)		
Hepatomegaly	2 (4.8)		
Pericarditis	1 (2.4)		
Pleuritis	6 (14.3)		
Arthralgia	23 (54.8)		
Arthritis	13 (31.0)		
Hemoglobin, g/dL	12.0 ± 1.8	13.0 ± 1.6	
Leukocyte, /μL	13,519 ± 10,169	6,090 ± 2,207	
Neutrophil, /μL	11,395 ± 10,151	3,707 ± 2,075	
Platelet, x10^3/μL	287.6 ± 126.1	255.3 ± 60.0	
Ferritin, ng/mL	3,708.1 ± 5,973.1	325.8 ± 348.3	
ESR, mm/hr	46.8 ± 29.7	20.7 ± 22.8	
CRP, mg/dL	8.21 ± 9.74	0.66 ± 1.92	
AST, mg/dL	83.7 ± 172.2	25.4 ± 14.5	
ALT, mg/dL	109.4 ± 240.6	21.2 ± 18.3	
Bilirubin, mg/dL	0.62 ± 0.48	0.59 ± 0.28	
Albumin, g/dL	4.01 ± 0.55	4.44 ± 0.58	
Systemic score	3.48 ± 2.43		
DAS-28		2.72 ± 1.37	

AOSD, adult-onset Still's disease; RA, rheumatoid arthritis; HC, healthy control; F/M, female/male; ESR, erythrocyte sedimentation rate; CRP, C-reactive protein; AST, aspartate transaminase; ALT, alanine transaminase; DAS-28, Disease Activity Score including 28 joints

*Matched patient characteristics

Supplementary Table 2. Linear regression analysis of CCR2 and CCL2 levels with disease activity markers and clinical manifestations in patients with adult-onset Still's disease.

Disease activity markers	β (p-value)	
	CCR2	CCL2
Systemic score	0.19 (0.183)	0.316 (0.024)
Hemoglobin	-0.09 (0.368)	-0.236 (0.018)
Leukocyte	0.417 (<0.001)	0.316 (0.041)
Platelet	0.036 (0.722)	-0.169 (0.09)
ESR	0.239 (0.016)	0.242 (0.015)
CRP	0.27 (0.006)	0.146 (0.145)
Ferritin	0.147 (0.294)	0.437 (0.001)
LDH	0.199 (0.156)	0.267 (0.055)
Albumin	-0.039 (0.697)	-0.062 (0.541)
Bilirubin	0.05 (0.624)	-0.044 (0.661)
AST	0.07 (0.485)	0.225 (0.024)
ALT	0.19 (0.183)	0.181 (0.07)
Fever	0.205 (0.149)	0.304 (0.03)
Sore throat	-0.065 (0.650)	-0.065 (0.653)
Skin rash	-0.014 (0.920)	0.2 (0.16)
Lymphadenopathy	-0.001 (0.997)	0.229 (0.106)
Splenomegaly	0.120 (0.402)	0.013 (0.929)
Hepatomegaly	-0.082 (0.566)	0.043 (0.766)
Pericarditis	-0.061 (0.672)	0.043 (0.766)
Pleuritis	0.261 (0.064)	0.027 (0.85)
Arthralgia	0.063 (0.66)	-0.162 (0.257)
Arthritis	0.253 (0.073)	-0.086 (0.55)

CCR, C-C motif chemokine receptor; CCL, C-C motif ligand; ESR, erythrocyte sedimentation rate; CRP, C-reactive protein; LDH, lactate dehydrogenase; AST, aspartate transaminase; ALT, alanine transaminase.

Supplementary Table 3. Comparison of CCR2 and CCL2 levels according to disease manifestations in adult-onset Still's disease patients.

Manifestations		CCR2, ng/mL	*p*-value	CCL2, pg/mL	*p*-value
Fever	(+), n = 30	84.78 ± 166.26	0.554	591.95 ± 786.11	0.003
	(-), n = 12	37.50 ± 111.90		187.55 ± 114.44	
Sore throat	(+), n = 14	46.24 ± 112.44	0.742	367.17 ± 233.56	0.272
	(-), n = 28	83.78 ± 170.07		531.03 ± 827.92	
Skin rash	(+), n = 27	59.03 ± 133.36	0.47	550.61 ± 755.35	0.019
	(-), n = 15	93.29 ± 186.08		342.84 ± 548.78	
Lymphadenopathy	(+), n = 10	60.78 ± 179.63	0.738	722.78 ± 1039.78	0.079
	(-), n = 32	74.55 ± 146.64		399.42 ± 537.02	
Splenomegaly	(+), n = 4	4.07 ± 2.19	1.0	460.18 ± 378.37	0.371
	(-), n = 38	78.34 ± 159.26		478.12 ± 717.54	
Arthritis	(+), n = 13	121.16 ± 196.64	0.36	341.13 ± 264.83	0.957
	(-), n = 29	48.91 ± 126.43		537.04 ± 808.02	

CCR, C-C motif chemokine receptor; CCL, C-C motif ligand.

Supplementary Table 4. Follow-up sample data of patients with adult-onset Still's disease (n = 7).

	Active status	Inactive status	P-value
CCR2, ng/mL	134.54 ± 200.41	11.34 ±11.78	0.159
CCL2, pg/mL	472.46 ± 280.11	223.85 ± 53.36	0.029
Leukocyte, /μL	13,871 ± 4,074	6,100 ± 2,139	0.003
ESR, mm/hr	48.43 ± 20.08	4.57 ± 4.08	0.001
CRP, mg/dL	7.33 ± 4.93	0.07 ± 0.04	0.008
Ferritin, ng/mL	4,294.3 ± 6,134.3	103.6 ± 74.4	0.123
LDH, U/L	592.1 ± 701.7	211.4 ± 29.5	0.203
Albumin, g/dL	3.74 ± 0.42	4.73 ± 0.26	0.003
AST, U/L	186.6 ± 379.2	17.1 ± 3.6	0.283
ALT, U/L	231.0 ± 425.2	13.9 ±6.4	0.228
Systemic score	4.14 ± 1.07	0.0 ± 0.0	<0.001

CCR, C-C motif chemokine receptor; CCL, C-C motif ligand; ESR, erythrocyte sedimentation rate; CRP, C-reactive protein; LDH, lactate dehydrogenase; AST, aspartate transaminase; ALT, alanine transaminase.

Supplementary Figure 1. Downstream signaling pathway of CCL2-CCR2 axis through stimulation of AOSD NETs.

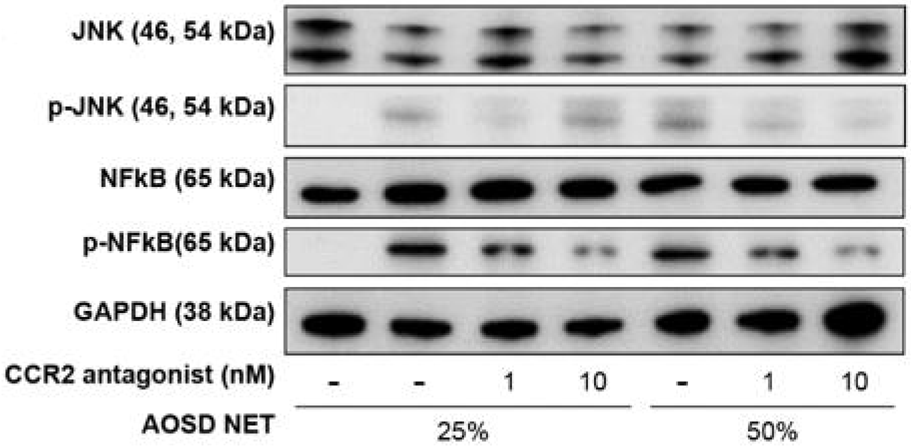

Supplementary Figure 2. Western blot original files.

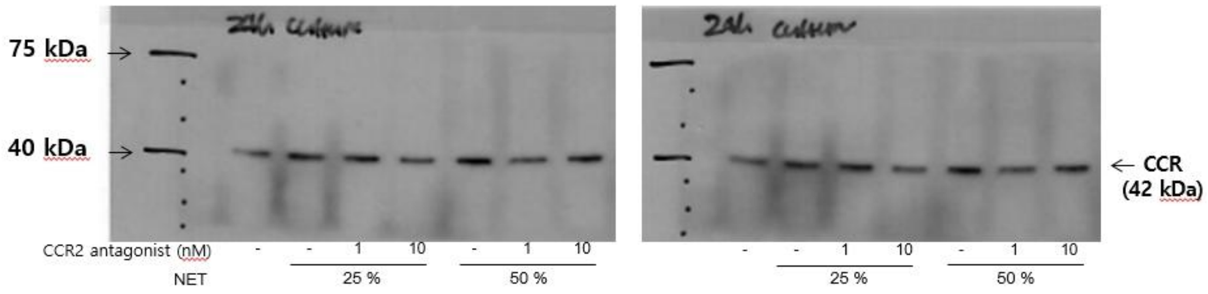

The western blotting film was taken twice with different exposure times.

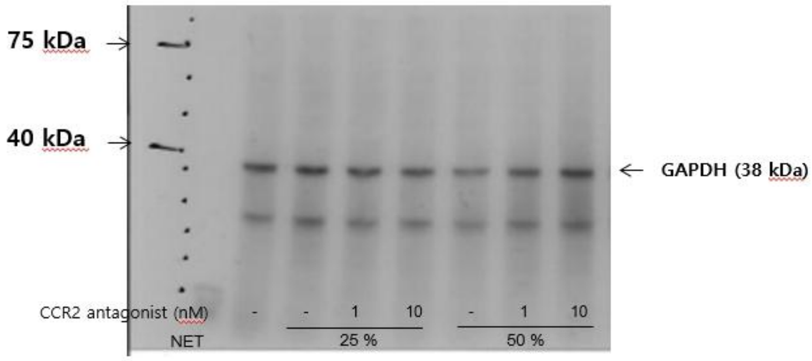

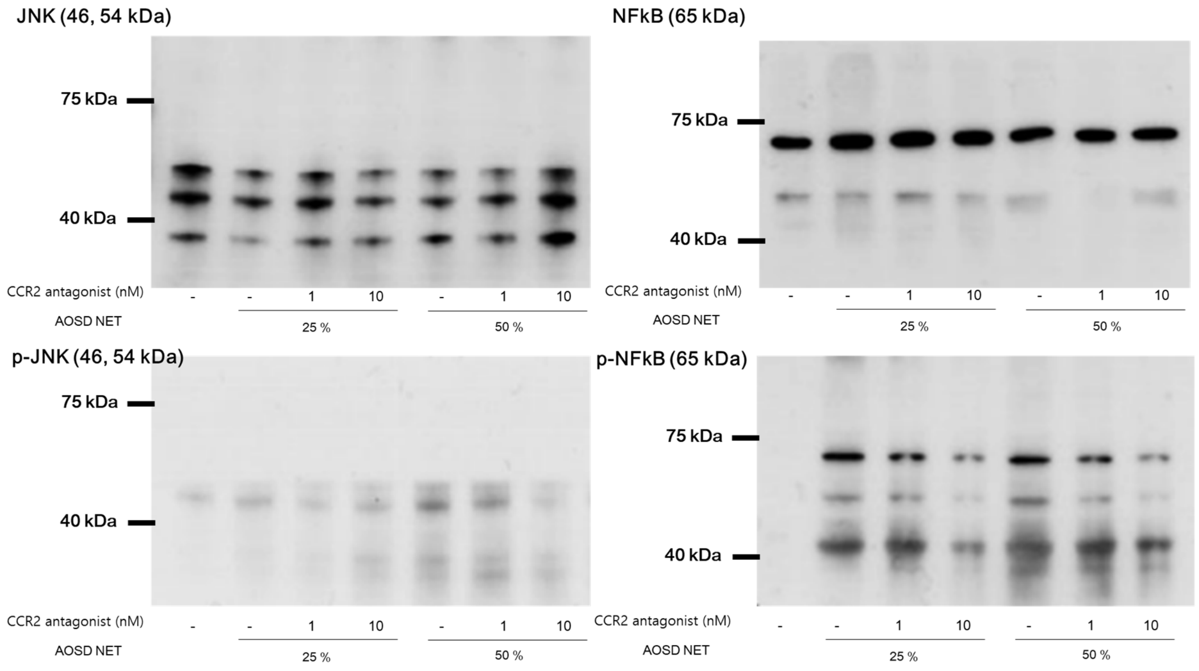

Supplement: Supplementary file 1 — Supplementary Information. [file 41598_2023_39517_MOESM1_ESM.pdf]
